# High prevalence of HPV 18 and multiple infections with oncogenic HPV genotypes in women at risk of cervical cancer examined in Manaus, Brazil

**DOI:** 10.1590/1414-431X2023e12720

**Published:** 2023-05-29

**Authors:** C. Fantin, J.B. Freitas, H.F.M. Teles, B.A.S. Oliveira, D.V. Brito

**Affiliations:** 1 Programa de Pós-graduação em Biotecnologia e Recursos Naturais da Amazônia, Escola Superior de Ciências da Saúde, Universidade do Estado do Amazonas, Manaus, AM, Brasil; 2 Laboratório de Genética Humana, Escola Superior de Ciências da Saúde, Universidade do Estado do Amazonas, Manaus, AM, Brasil; 3 Departmento de Ginecologia e Obstetrícia, Escola Superior de Ciências da Saúde, Universidade do Estado do Amazonas, Manaus, AM, Brasil; 4 Policlínica João dos Santos Braga, Secretaria de Estado de Saúde do Amazonas, Manaus, AM, Brasil

**Keywords:** Human papillomavirus, High-risk genotypes, Cervical lesions, Colposcopic examination, Multiple infections

## Abstract

Cervical cancer is a serious public health problem in Brazil, especially in Manaus (Amazonas), the city with the highest incidence rate of cervical cancer in the country. Persistent infection with oncogenic human papillomavirus (HPV) genotypes is the cause of disease development. The aim of this study was to investigate the prevalence of oncogenic genotypes in women at high risk for cervical precancer examined in two policlinics in Manaus. One hundred and two patients who underwent colposcopy took part in the research. The DNA samples obtained from the cervical epithelium were analyzed by PCR with type-specific primers for the detection of eight oncogenic genotypes, which were chosen based on previous studies. The presence of HPV virus was detected in all samples. The most prevalent oncogenic genotypes were 18 (47.1%) and 16 (45.1%). Interestingly, HPV 18 was considered uncommon in this region. In addition to these, genotypes 31 (19.6%), 58 (19.6%), 33 (18.6%), and 45 (15.7%) also had a relatively high frequency in this population. Fifty-six women (54.9%) had multiple infections with up to five oncogenic types. Also, the presence of genotypes other than 16 and 18 was observed in most samples (57.8%), which also deserves attention since they are not covered by currently available vaccines against HPV in Brazil. The high prevalence and multiple infections with several oncogenic HPV genotypes in association with precursor lesions for cervical cancer highlighted the need to improve strategies to prevent this disease in Amazonas.

## Introduction

Cervical cancer (CC) is a serious public health problem due to its high incidence and mortality rate in the female population. CC is the second most incident cancer in the state of Amazonas (27.60/100,000) after non-melanoma skin cancer, with the number of cases doubling in the city of Manaus (51.64/100,000) (1). The main factor leading to the development of CC is human papillomavirus (HPV) infection.

HPV is an epitheliotropic virus belonging to the family Papillomaviridae and is the most common sexually transmitted pathogen in the world. These viruses can be classified into different genotypes based on the percentage of similarity in the nucleotide sequence of the L1 gene, which is the most conserved region of the viral genome (2). More than 200 HPV genotypes have already been described (3). According to epidemiological criteria, HPV genotypes can be classified as low risk, which are associated with the development of genital warts, and high risk, which are often associated with CC. Approximately 15 genotypes are classified in the high-risk category and three in the probable high-risk category, which are also known as oncogenic genotypes (4).

It is estimated that a large part of sexually active women will be exposed to HPV. Most infections are cleared by the immune system within two years after exposure (5). The persistence of oncogenic types, however, leads to the development of CC. Cancer progression occurs through significant transformations of the epithelial tissue of the cervix, ranging from precancerous lesions to the invasive stage (3). This progression occurs asymptomatically, thus hindering detection. However, if detected early, almost all lesions can be cured (6).

To fight CC, primary and secondary prevention measures have been adopted worldwide. Primary prevention is about reducing the risk of HPV infection, and the main option is vaccination (7). In Brazil, the vaccine that covers types 6, 11, 16, and 18 (quadrivalent) began to be distributed in 2014 (8). Although the quadrivalent vaccine covers the main HPV types prevalent worldwide, it is important to observe whether the vaccine used is actually covering the genotypic diversity of each region (9). Secondary prevention, on the other hand, is related to screening strategies for CC, and is carried out mostly through diagnostic tests aimed at verifying the presence of precursor lesions for cancer (also called precancerous lesions or cervical precancer), so they can be treated before they become an invasive carcinoma. In case of abnormal findings with a significant risk of precancerous lesions on the cervical cytology screening, follow-up colposcopy is performed to provide a more thorough investigation of the cervix and, if required, histology examination is also performed, which is the reference diagnosis standard to confirm the presence of precancerous lesions and guide management and treatment decisions (10).

More recently, the World Health Organization and European guidelines have recommended the use of HPV DNA testing as the primary method in screening programs (11,12). This method is highly sensitive, objective, and reproducible, overcoming the issues with poor reproducibility and equivocal findings of the cytological exam. In such programs, cytology is used only as a triage test for positive HPV results (13). Currently, HPV testing is successfully used as primary screening in some European countries (14). However, the switch from the existing cytology-based screening to a new HPV-based screening is a major challenge, especially in low- and middle-income countries. Thus, local conditions of different geographical regions should be considered in future transitions (13,15).

Moreover, HPV testing has become an important tool in epidemiological studies, since it allows the investigation of the frequency of circulating genotypes in a given location (16). Two major molecular methods are used for the detection of HPV DNA: direct hybridization and different types of polymerase chain reaction (PCR) (17). In PCR-based methods, the use of specific primers for different HPV genotypes has the advantages of greater sensitivity, allowing the identification of multiple HPV infections, and cost-effectiveness (16).

In view of the above, the present study investigated the prevalence of oncogenic HPV genotypes in women from Manaus at risk of precancerous cervical lesions. We found HPV 18 as the most prevalent genotype, which was previously considered uncommon there. Also, the concomitant presence of two or more genotypes in most infected women might be contributing to the high risk of cervical cancer development in this region.

## Material and Methods

### Patient profile and study environment

The participants of this study were patients examined at the João dos Santos Braga and Governador Gilberto Mestrinho Outpatient Clinics, located in the city of Manaus, Amazonas, Brazil, from January 2019 to December 2020. These policlinics are recognized as reference centers for colposcopies and receive patients from several locations in the state of Amazonas through the National Regulatory System (SISREG).

Women who presented cytological alterations in cytology tests and who were referred for a colposcopy examination according to the National Screening Guidelines for Cervical Cancer (18) were invited to participate in the research through an interview. The inclusion criteria were sexually active women aged 25 to 64 years who presented the following abnormalities in cytological findings (in decreasing order of risk): high-grade squamous intraepithelial lesion (HSIL); atypical squamous cells, cannot exclude HSIL (ASC-H); and two consecutive diagnoses of low-grade squamous intraepithelial lesion (LSIL). This classification is according to the Bethesda system, and these categories indicate the possibility of underlying immediate precancerous lesions, which is especially high in HSIL cases (10). The exclusion criteria were pregnant women with obstetric restrictions, women who had had a hysterectomy or conization, women with vaginal bleeding, and menopausal women with cervical atrophy at the time of the examination. During the interview with the participants, we also asked if they had been vaccinated against HPV.

This study was part of the project approved by the Research Ethics Committee of the State University of Amazonas under the license number 4.288.698. A written informed consent was obtained from all participants. The information regarding the result of cytology examination was provided by the patients or verified in their medical record.

### Sample collection and DNA extraction

Samples of the cervical epithelium from 102 patients selected for the study were collected by a healthcare professional with a sterile cervical brush (Kolplast^®^, Brazil), before the start of the colposcopy examination. The brush containing the cervical scraping was inserted into a 1.5-mL microtube containing 500 µL of TE buffer (10 mM Tris-HCl pH 8.0 and 1 mM EDTA). The collected samples were sent to the Laboratory of Human Genetics of the Amazonas State University (UEA), where they were stored at -20°C.

Total DNA extraction was performed according to the CTAB method (19), which was adapted to cells of the cervical epithelium. In this adapted protocol, the microtubes containing the cervical scraping in TE buffer were centrifuged (4,300 *g*, 5 min, room temperature) for the recovery of the cell pellet before the lysis step with the CTAB detergent.

### PCR for HPV genotyping

The determination of HPV genotypes was carried out using the PCR method in three steps: 1) detection of human genomic DNA (to determine the DNA quality of the samples); 2) detection of HPV-DNA; and 3) detection of specific genotypes.

The first step was performed with primers for the human β-globin gene (20). In the second reaction, GP5+/6+ primers were used to amplify the HPV L1 gene (21). The protocol used for PCR reaction was adapted from Silva et al. (22), containing the following components: 1× buffer, 1.5 mM MgCl_2_, 0.2 mM dNTPs, 0.1 µM of each primer, 0.5 U Taq, and 150 ng total DNA. PCR conditions were initial denaturation at 95°C for 4 min, followed by 40 cycles at 95°C for 1 min, 57°C for 1 min, and 72°C for 1 min, and final extension at 72°C for 5 min.

In the third step, all positive samples for HPV-DNA (primers GP5+/6+) were tested for the detection of eight oncogenic HPV types using specific primers (Table 1). The genotypes were chosen based on the HPV molecular detection studies previously carried out in the Brazilian Amazon region (23-25). The reaction and cycling conditions for each genotype were the same as previously described for HPV-DNA detection, except for the annealing temperatures (26-28, Table 1). In all steps, the final volume of the reaction was 15 µL.

**Table 1:** Sequences of the specific primers used for the detection of the eight oncogenic HPV genotypes analyzed in this study.

Genotype	Sequences	Size	AT
HPV 16^1^	F: 5'-TTGCAGATCATCAAGAACACGTAGA-3'	111 bp	57°C
	R: 5'-GTAGAGATCAGTTGTCTCTGGTTGC-3'		
HPV 18^1^	F: 5'-CAACCGAGCACGACAGGAACG-3'	172 bp	56°C
	R: 5'-TAGAAGGTCAACCGGAATTTTCAT-3'		
HPV 31^2^	F: 5'-GAAATTGCATGAACTAAGCTCG-3'	263 bp	54°C
	R: 5'-CACATATACCTTTGTTTGTCAA-3'		
HPV 33^2^	F: 5'-ACTATACACAACATTGAACTA-3'	398 bp	58°C
	R: 5'-GTTTTTACACGTCACAGTGCA-3'		
HPV 45^2^	F: 5'-GTGGAAAAGTGCATTACAGG-3'	151 bp	56°C
	R: 5'-ACCTCTGTGCGTTCCAATGT-3'		
HPV 52^2^	F: 5'-TAAGGCTGCAGTGTGTGCAG-3'	229 bp	54°C
	R: 5'-CTAATAGTTATTTCACTTAATGGT-3'		
HPV 53^3^	F: 5'-TTGTTCAGTGTACGGGGCTAGC-3'	549 bp	61°C
	R: 5'-GTGACGCCATTGCAGTTATCGCCT-3'		
HPV 58^3^	F: 5'-GTAAAGTGTGCTTACGATTGC-3'	274 bp	62°C
	R: 5'-GTTGTTACAGGTTACACTTGT-3'		

AT: annealing temperature. ^1^Swan et al. (26); ^2^Sotlar et al. (27); ^3^Romero-Pastrana (28).

For the detection of specific genotypes, a previously identified sample was added to each reaction as a positive control for the presence of the analyzed genotype, in addition to a blank control. The positive controls were provided by the Molecular Diagnostic Laboratory at the Federal University of Amazonas. The presence or absence of each genotype was verified through the analysis of PCR products by electrophoresis in 1.5% agarose gel stained in GelRed^TM^ (Biotium^®^, USA).

### Data analysis

Frequency, mean, and standard deviation were calculated for age groups (25-35, 36-45, 46-53, and ≥54) using the Biostat 5.3 program (https://www.analystsoft.com/en/products/biostat/). In the analysis of the HPV genotyping data, the absolute frequency and relative frequency (in percentage) for each genotype were first verified in relation to the total number of samples. The percentage of multiple infections (concomitant presence of more than one genotype) and the frequency of viral genotypes in relation to the total number of genotypes were also calculated. Finally, the relationship between cytological abnormalities and HPV genotypes was observed by calculating the frequencies of genotypes found in each category.

## Results

One hundred and two women agreed to participate in the study. The overall mean age was 43 years (SD=12.01). In relation to the age groups, there was a predominance of women aged 36 to 45 years (n=31; 30.39%), closely followed by women aged 24 to 35 years (n=29; 28.2%). As for place of birth, 41 women (40.2%) were from Manaus, 45 (44.1%) were from municipalities of inland Amazonas, and 16 (15.7%) came from other states of Brazil but were living in Manaus. All participants reported that they were not vaccinated against HPV.

### Prevalence of HPV genotypes

In this study, all samples (n=102) were positive for the amplification of the β-globin gene, confirming the DNA quality of the specimens. Moreover, the presence of HPV-DNA was confirmed in all samples via amplification of the L1 gene.

**Figure 1:**
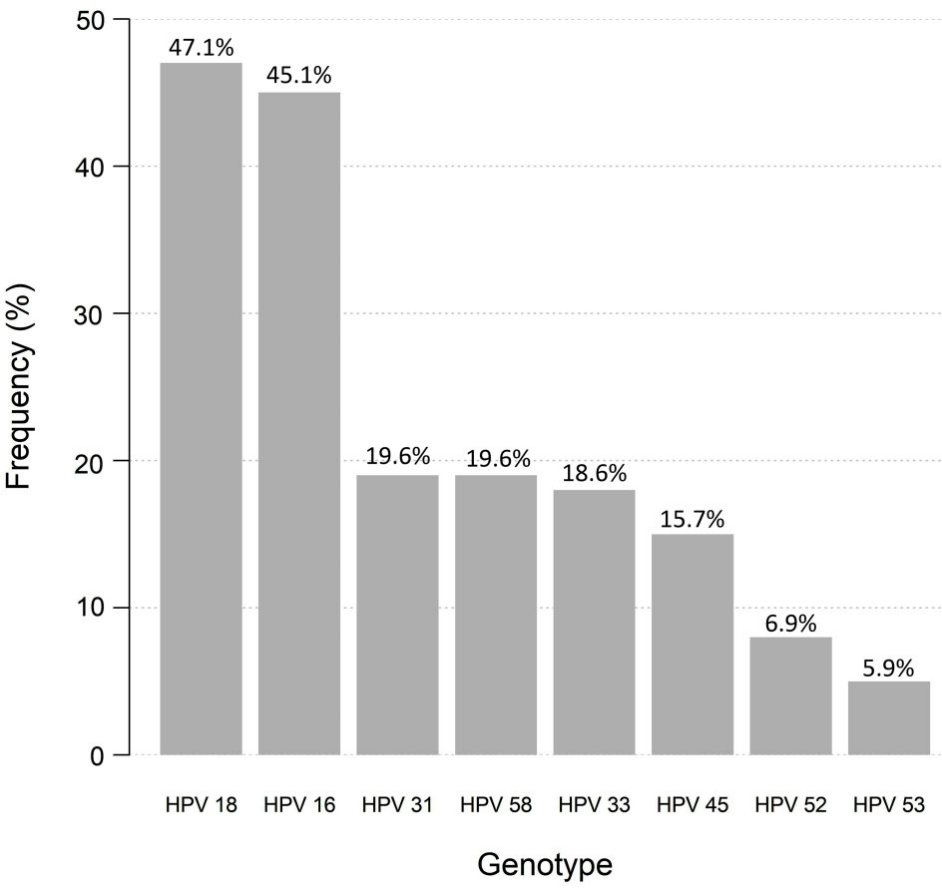
Relative frequency (%) of eight high-risk HPV genotypes detected in patients examined for precursor lesions for cervical cancer in Manaus.

Regarding the analysis of the HPV genotypes, it was possible to detect the presence of oncogenic genotypes in the analysis of most samples (84.3% or 86/102). The frequencies found for the genotypes were: HPV 18 (48/102; 47.1%), 16 (46/102; 45.1%), 31 (20/102; 19.6%), 58 (20/102; 19.6%), 33 (19/102; 18.6%), 45 (16/102; 15.7%), 52 (7/102; 6.9%), and 53 (6/102; 5.9%) (Figure 1).

**Figure 2:**
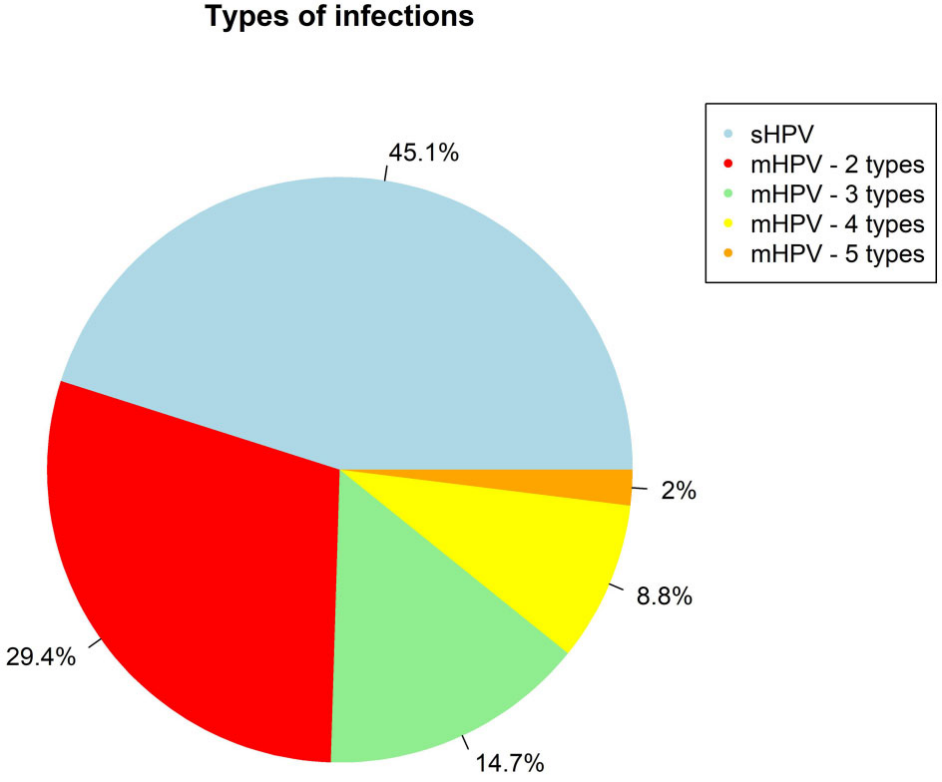
Relative frequency (%) of single (sHPV) and multiple (mHPV) infections of HPV genotypes detected in patients with precursor lesions for cervical cancer and examined in Manaus.

The presence of more than one type of HPV (multiple infection or mHPV) was observed in 54.9% (56/102) of women, and up to five viral types were detected concomitantly. Among these cases, multiple infections by two genotypes were detected in 29.4% (30/102) of the samples; by three genotypes in 14.7% (15/102); by four genotypes in 8.8% (9/102); and by five genotypes in 1.9% (2/102) (Figure 2). The eight genotypes analyzed were observed in these combinations. The most predominant genotypes among multiple infections were HPV 16 (37/56; 66.1%), 18 (36/56; 64.3%), 58 (19/56; 33.9%), 31 (16/56; 28.6%), and 33 (14/56; 25.0%). The most frequently observed combination of genotypes was the coinfection of 16 and 18 (9/56; 16.1%).

Considering single and multiple infections, the two most common genotypes worldwide, HPV 16 and 18, were observed in 65.7% (67/102) of participants, while the other six oncogenic genotypes were detected in 57.8% (59/102).

### Relationship between cytological abnormalities and HPV genotypes

In the study of cytological abnormalities detected in the cytology test of the study participants, it was found that 49.0% (50/102) of the women had a diagnosis of HSIL; 33.3% (34/102) of ASC-H; and 17.7% (18/102) of LSIL. Genotypes 16 and 18 were the most frequent in the three categories. In women with HSIL, genotypes 31 (18.0%), 33 (18.0%), and 58 (18.0%) followed 16 and 18; in women with ASC-H, genotypes 33 (20.6%) and 31 (17.6%) were the next ones; and in women with LSIL, genotypes 45 (33.3%) and 58 (33.3%) were in third place (Table 2). It should be noted that multiple infections were considered to estimate the most frequent genotypes, and this type of infection was detected in 58% (29/50) of women with an HSIL diagnosis, 55.5% (10/18) with an LSIL diagnosis, and 50% (17/34) with an ASC-H diagnosis. Women that were HPV-positive, but negative for these eight genotypes, comprised 29.4% (10/34) of the ASC-H group, 16.7% (3/18) of the LSIL group, and 6% (3/50) of the HSIL group.

**Table 2:** Frequency of HPV genotypes in groups of cytological abnormalities in the participants of this study.

Viral types	HSIL (n=50)		ASC-H (n=34)		LSIL (n=18)	
	AF	%	AF	%	AF	%
16	24	48.0	13	38.2	8	44.4
18	24	48.0	16	47.1	7	38.8
31	9	18.0	6	17.6	4	22.2
33	9	18.0	7	20.6	2	11.1
45	6	12.0	5	14.7	6	33.3
52	3	6.0	4	11.8	2	11.1
53	1	2.0	1	2.9	3	16.6
58	9	18.0	4	11.8	6	33.3
Total	85		56		38	

Total absolute frequencies of genotypes within each category are higher than the number of samples due to the number of multiple infections. HSIL: high-grade squamous intraepithelial lesion; ASC-H: atypical squamous cells that cannot exclude HSIL; LSIL: low-grade squamous intraepithelial lesion; AF: absolute frequency.

## Discussion

In this study, we estimated the frequency of circulating oncogenic HPV genotypes in women with cytological abnormalities. Similar studies on the molecular detection of HPV virus in women with cytological abnormalities, including high- and low-grade intraepithelial lesions in Manaus, have been performed previously (22,23,29). Here, however, emphasis was placed on obtaining a larger sample of cytological abnormalities referred to colposcopy, as they present greater risk for the development of cancer. Other studies in the northern region of Brazil focused on specific populations of participants, such as indigenous women and HIV-positive women (25,30,31). In addition, a technique with high sensitivity (PCR with specific primers) was used for the individual detection of eight oncogenic genotypes and visualization of possible multiple infections.

All the samples analyzed here were positive for HPV-DNA, which is consistent with the severity of lesions referred for colposcopy. Other studies conducted in the state of Amazonas using molecular tests showed a prevalence of HPV infection ranging from 6.5% (29) to 39.7% (30) in normal samples and 38.5% (29) to 93.6% (23) in women with cytological abnormalities in general.

Most of our samples (84.3%) were positive for at least one of the eight genotypes analyzed. Six oncogenic types were observed at a relevant frequency (above 15%): 18, 16, 31, 58, 33, and 45, in descending order. Genotype 18 was the most prevalent in our analyses, different from that expected, since the frequency of HPV 18 was previously considered low and not very relevant in Amazonas based on previous studies. Silva et al. (22) analyzed the presence of HPV 16 and 18 in patients who were referred for colposcopy examination in Manaus and found a much higher frequency of genotype 16 (59.2%) compared to their findings for genotype 18 (8.2%). In the studies of Castro et al. (29) and Costa-Lira et al. (23), also in Manaus, HPV 16 was the most frequent genotype in samples with high- and low-grade lesions (HSIL and LSIL), followed by genotype 58, while HPV 18 was not found in any of the samples analyzed by those authors. In addition, Rocha et al. (24), who analyzed groups of women diagnosed with normal and altered cytology in Coari city, observed a high prevalence of genotype 16 and absence of HPV 18 also in this population. These studies, however, used the Sanger sequencing method to identify genotypes, which can only detect the HPV genotype that is present in a greater quantity in the sample. Therefore, the difference between the methods used for HPV detection may be related to the different results observed. This shows the importance of using PCR for specific genotypes, since it is a tool with an optimal cost-benefit ratio for epidemiological studies.

In this study, multiple HPV infections were detected in 54.9% (56/102) of participants, which was higher than expected given the limited number of genotypes analyzed. Resende et al. (32) observed the occurrence of 52% multiple infections in patients from the Midwest and Southeast of Brazil with cytological alterations in general, using a method for the detection of a higher number of genotypes than this one (reverse hybridization with probes for 21 high-risk genotypes and 16 low-risk genotypes). These authors found that the most prevalent co-infection in their study was HPV 16/58 (12.7%), unlike here, in which the most prevalent co-infection was HPV 16/18 (16%). Also, Fonseca et al. (30) observed the concomitant presence of oncogenic genotypes in a study with opportunistic screening in indigenous women of two states of the Brazilian Amazon region (Roraima and Amazonas). They used next-generation sequencing, a sensitive method but with high cost. These authors detected multiple infections in 45.1% of HPV-positive samples, and up to 11 genotypes were identified per sample. De Brot et al. (33) proposed that multiple infections by oncogenic genotypes are associated with a higher risk of persistent HPV infection and progression to high-grade lesions. Recently, Oyervides-Muãoz et al. (34) proposed that multiple infections be considered a marker for the identification of patients with a higher risk of precancerous lesions and progression to CC. These findings demonstrate the importance of giving attention to this high prevalence of multiple HPV infections.

Moreover, the results observed here revealed that, when multiple infections were considered, more than half of the participants (57.8%) were infected with genotypes other than 16 and 18, which are not covered by the vaccine used in Brazil (8). This frequency is remarkably high compared to a similar study performed in Northeast Brazil (Agreste region) in women with abnormal cervical histopathological diagnosis, which found 27% of patients infected with HPV other than 16 and 18 (35). These findings have major implications for local public health policies, especially primary prevention strategies, and corroborate the need to identify the genotypic HPV diversity at the regional level.

As for the relationship between cytological abnormalities (HSIL, ASC-H, and LSIL) and genotype detection, HPV 18 was the most prevalent in all groups, followed by HPV 16. In the next positions, the frequency of HPV 31 and 33 was highlighted in HSIL and ASC-H; HPV 58, in HSIL and LSIL; and HPV 45, in LSIL samples. These cytological findings can be either confirmed or excluded with follow-up colposcopy and cervical biopsy. However, the risk for precursor lesions is significantly higher in women that are positive for oncogenic genotypes (36), which was the case of most patients in this study (83.3%). Thus, our findings confirmed the high risk of CC development for most patients that were referred for colposcopy examination and the importance of HPV genotyping for a closer follow-up.

In conclusion, the eight oncogenic HPV genotypes analyzed circulate in the population of Manaus and are associated with women at risk of cervical precancer. HPV 18 was the most frequent in our study, contrary to expectations, since it was previously considered uncommon in the region. In addition to HPV 16 and 18, the other high-risk genotypes that require special attention in this population are HPV 31, 33, 45, and 58. The presence of multiple infections in half of the group of women analyzed was also higher than expected. It is important that these issues continue to be investigated in future studies. The predominance of HPV 16 and 18 and the presence of multiple infections with oncogenic genotypes are indicators of a high risk of emergence of new cases of CC in this population, which highlights the need to increase the effectiveness of primary prevention and improve CC screening programs in Amazonas.
